# Growth and eGFP Production of CHO-K1 Suspension Cells Cultivated From Single Cell to Laboratory Scale

**DOI:** 10.3389/fbioe.2021.716343

**Published:** 2021-10-15

**Authors:** Julian Schmitz, Oliver Hertel, Boris Yermakov, Thomas Noll, Alexander Grünberger

**Affiliations:** ^1^ Multiscale Bioengineering, Faculty of Technology, Bielefeld University, Bielefeld, Germany; ^2^ Center for Biotechnology (CeBiTec), Bielefeld University, Bielefeld, Germany; ^3^ Cell Culture Technology, Faculty of Technology, Bielefeld University, Bielefeld, Germany

**Keywords:** microfluidics, single-cell cultivation, single-cell analysis, miniaturization, scale comparison, cell culture, GFP production

## Abstract

Scaling down bioproduction processes has become a major driving force for more accelerated and efficient process development over the last decades. Especially expensive and time-consuming processes like the production of biopharmaceuticals with mammalian cell lines benefit clearly from miniaturization, due to higher parallelization and increased insights while at the same time decreasing experimental time and costs. Lately, novel microfluidic methods have been developed, especially microfluidic single-cell cultivation (MSCC) devices have been proved to be valuable to miniaturize the cultivation of mammalian cells. So far, growth characteristics of microfluidic cultivated cell lines were not systematically compared to larger cultivation scales; however, validation of a miniaturization tool against initial cultivation scales is mandatory to prove its applicability for bioprocess development. Here, we systematically investigate growth, morphology, and eGFP production of CHO-K1 cells in different cultivation scales ranging from a microfluidic chip (230 nl) to a shake flask (125 ml) and laboratory-scale stirred tank bioreactor (2.0 L). Our study shows a high comparability regarding specific growth rates, cellular diameters, and eGFP production, which proves the feasibility of MSCC as a miniaturized cultivation tool for mammalian cell culture. In addition, we demonstrate that MSCC provides insights into cellular heterogeneity and single-cell dynamics concerning growth and production behavior which, when occurring in bioproduction processes, might severely affect process robustness.

## Introduction

The number of biotechnologically manufactured products like biopharmaceuticals has increased rapidly over the last decades (Walsh, 2018). Consequently, there is a continuing desire for new and more efficient bioprocesses to cover the increasing demand. Lately, the development of improved bioprocesses went hand in hand with the technological progress of miniaturization (Hemmerich et al., 2018). Since the first approach, the focus of scale-down applications lies on the same ambitions: to reduce experimental time and simultaneously increase insights (Kim et al., 2012; Janakiraman et al., 2015). Given that mammalian cell culture processes require considerably longer experimental time spans than bacterial processes, and process development is often based on empirical testing of multiple interdependent parameters (Neubauer et al., 2013), especially time reduction and increasing experimental throughput are highly desirable to enhance process development (Zhang et al., 2010; Rameez et al., 2014). Furthermore, maximizing the analytical throughput and expanding the degree of parallelization improve not only process development but also cell line or medium design (Betts and Baganz, 2006).

Miniaturizing a bioproduction process often depends on novel bioreactor concepts that do not match the original cultivation conditions or cultivation vessel geometry of the manufacturing scale, which makes systematic validation mandatory. Therefore, to qualify a technology for miniaturization, the recorded data need to be verified against data from original scale approaches (Li et al., 2006; Tsang et al., 2014). If the generated data are not comparable, prediction for the original scale based on the data from the miniaturized scale will be unfeasible and ultimately will lead to deviations in process development or challenges in eventual scale-up (Betts and Baganz, 2006; Bertrand et al., 2018).

Lately, different approaches to miniaturize mammalian cultivation for bioprocess development and screening have been introduced, all based upon different concepts ranging from shake flask applications to shaken microtiter plates and miniaturized stirred bioreactors (Zhang et al., 2010). An already established miniaturized stirred bioreactor consists of the Ambr™ platform, which proved suitable to emulate temperature, dissolved oxygen, and pH profiles matching large-scale bioreactors and shows comparable growth and productivity (Rameez et al., 2014). Applying the 300-µl chamber–based SimCell micro-bioreactor system, Legmann et al. (2009) showed high comparability between a micro-bioreactor and 3-L benchtop bioreactors and expanded the experimental results by high-throughput multifactorial experiments at the same time. Using a shaken 24-well single-use cassette with bubble columns, Betts et al. (2014) were able to mimic industrially relevant fed-batch processes with comparable growth and production performance. Furthermore, orbitally shaken tubes have been applied as a miniaturization tool to optimize operating conditions of mammalian perfusion cultures and showed good comparability with benchtop bioreactors (Wolf et al., 2018). Besides already established approaches, microfluidic cultivation tools have become increasingly relevant in terms of downsizing and mark the next level of miniaturization (Mehling and Tay, 2014; Marques and Szita, 2017).

On the one hand, microfluidic approaches can be applied to investigate one discrete bioprocess-related question; on the other hand, microfluidics can be applied to miniaturize the whole bioprocess (Bjork et al., 2015). In addition to the mentioned preferences of miniaturization, microfluidics additionally extends the toolbox of already established approaches by the feature to cultivate and analyze cells with single-cell resolution (Hung et al., 2005; Lindström and Andersson-Svahn, 2010; Kolnik et al., 2012). Therefore, microfluidic single-cell tools can be applied to analyze cellular heterogeneity which would stay masked by standard average measurements conventionally used in bioprocess research. Due to the genetic plasticity and origin of every industrial production cell line, utilized populations doubtlessly exhibit genetic or phenotypic heterogeneity (Barnes et al., 2003; Pilbrough et al., 2009) which has been ignored in bioprocess development over the last decades (Schmitz et al., 2019).

In the context of bioprocess research and development, microfluidic single-cell cultivation (MSCC) represents the tool of choice to investigate cellular behavior concerning heterogeneity in growth and morphology, proliferation, and productivity (Schmitz et al., 2019). In contrast to other single-cell analysis applications like flow cytometry or droplet microfluidics, MSCC combines features necessary for long-term cultivation under controlled environmental conditions, which are needed for bioprocess near reasearch, with analytical prospects like live cell imaging and thereby facilitate high spatio-temporal resolution of cellular behavior (Grünberger et al., 2014).

In this work, we present a comparative study with a focus on the growth and production of CHO-K1 cells at different scales, ranging from single-cell cultivation (230 nl) and shake flasks (125 ml) to benchtop stirred tank bioreactors (2.0 L). Lately, we introduced a platform for mammalian single-cell cultivation which, for the first time, enabled cultivation of mammalian suspension cell lines with single-cell resolution under process near environmental conditions (Schmitz et al., 2021). To approach the question if mammalian single-cell cultivation is feasible for miniaturizing cultivation for future bioprocess screening approaches, we here investigate possible differences in terms of growth behavior, cell morphology, and eGFP production. Furthermore, we give an example of how MSCC can enlarge the insights into single-cell dynamics.

## Materials and Methods

### Cell Culture and Medium

In this work, the CHO-K1 cell line ATCC CCL-61, adapted to growth in suspension, was applied as a model for other mammalian cell lines used in biotechnology. Furthermore, an eGFP-producing CHO-K1 pool was applied for comparative studies of production behavior between cultivation scales. Therefore, the same CHO-K1 cell line was transfected with a vector containing eGFP under the control of the endogenous HSPA5 promoter and puromycin resistance for selection (Supplementary Figure S1). Two weeks after transfection and cultivation with 8 μg/ml puromycin, the eGFP gene was randomly integrated into the genome and the heterogeneous cell pool was cryopreserved.

For cultivation, a instead of the commercially available medium (TCX6D, Xell, Germany), supplemented with 6 mM glutamine, was utilized. The pre-culture of eGFP-expressing CHO-K1 cells was further supplemented with 8 μg/ml puromycin, but the main cultivation was executed without selective pressure. Initial CHO cell culture was inoculated from a uniform working cell bank and cultivated at a temperature of 37°C, 5% CO_2_, 80% humidity, and 185 rpm (maximal deflection 50 mm) on an orbital shaker (ES-X, Kühner AG, Switzerland). The first passage was performed in 50-ml TubeSpin® Bioreactor 50 (TPP®, Switzerland) with a working volume of 15 ml, and subsequent passages were performed in 125-ml shake flasks (Flat Base, TriForest, United States) with a working volume of 60 ml. For reproducibility, the pre-culture was passaged in the exponential phase two or three times before starting any of the following cultivation experiments.

### Microfluidic Single-Cell Cultivation

Microfluidic single-cell cultivation was performed, as described previously (Schmitz et al., 2021). The employed PDMS–glass device was mounted onto an automated inverted microscope for phase contrast microscopy (Nikon Eclipse Ti2, Nikon, Germany). For seeding cells into the cultivation chambers, CHO-K1 cell suspension from the exponential growth phase with a cell density of 3 or 5 × 10^6^ cells/ml was manually flushed through the cultivation device until the cultivation chambers were filled with cells sufficiently. By moving the cell suspension back and forth through the adjacent supply channels, cells randomly enter the cultivation chambers. The controlled introduction of air bubbles into the device improves cell seeding; however, any residual air must be flushed out prior to the MSCC experiment (Probst et al., 2015). Next, fresh medium mixed with conditioned medium obtained from the exponential growth phase in a ratio of 1:1 to simulate substrate and metabolite situation in standard batch cultivations was constantly perfused through the supply channels by low-pressure syringe pumps (neMESYS, CETONI, Germany), with a flow rate of 2 μl/min. Constant cultivation conditions of 37°C and 5% CO_2_ were controlled by microscope incubator systems (Cage incubator and H201-K-FRAME GS35-M, OKO Touch, Okolab S.R.L., Italy). For the microscopic analysis of single cells, a 40× objective was applied, and relevant positions were analyzed every 20 min (NIS Elements AR 5.20.01 Software, Nikon Instruments, Germany).

### Shake Flask Cultivation

Shake flask cultivation was performed as triplicates in 125-ml shake flasks (Flat Base, TriForest, United States) with a cultivation volume of 60 ml, each inoculated at 5 × 10^5^ cells/ml from one 250 ml pre-culture to assure reproducibility. Cultivation temperature, CO_2_ atmosphere, and humidity were matching the pre-culture conditions. The shaking frequency and maximal deflection were unchanged as well. Every 12 h, samples for the analysis of growth, viability, and morphology were taken and measured using a CEDEX cell counter (Innovatis, Germany).

### Bioreactor Cultivation

Bioreactor cultivation was performed as duplicates in 2-L Biostat B-DCU stirred tank bioreactors (Sartorius AG, Germany), with a working volume of 1.5 L, inoculated at 5 × 10^5^ cells/ml. The cultivation temperature, pH value, and dissolved oxygen concentrations were controlled at 37°C, 7.2, and 40% of the air saturation. For pH control, a CO_2_/NaHCO_3_-based buffer system was applied; target DO was adjusted by increasing the respective airflow. The stirring speed was set to 150 rpm using a Rushton turbine.

### Growth Rate Analysis

As a key indicator for growth comparison, the specific growth rate µ_max_ of each cultivation was analyzed. For microfluidic single-cell cultivation, the cell number was determined every 12 h by analyzing time-lapse images. By offsetting the cultivation chamber’s volume of 3.2 × 10^–7^ ml against the number of cells inside, the enumerated cell number can be converted into viable cell density. To evaluate specific growth rates, cell densities from microfluidic cultivation and viable cell densities from shake flask and bioreactor cultivation were plotted against cultivation time semi-logarithmically to identify the relevant interval for µ_max_ determination. In the following, µ_max_ was determined graphically from the slope of the exponential growth phase of each plot using OriginPro (OriginPro 2020 9.7.0.188, OriginLab Corporation, United States).

### Cell Morphology Analysis

In order to compare cell morphology over scales, for microfluidic cultivation, cellular area (A) was determined manually using ImageJ 1.52p (Schindelin et al., 2012) and converted into cell volume by multiplying cell areas with the cultivation chamber height (h) of 8 μm, implying a cylindrical cellular shape inside the microfluidic device. Assuming a natural sphere-shaped cellular morphology without the restrictive height, calculated volumes were again converted into cellular diameter (d) data:

$$d=\sqrt[{3}]{\frac{3\cdot \left(A\cdot h\right)}{4\cdot \pi }}$$

To enlarge the sample size and thereby statistical significance, the number of analyzed cultivation chambers was increased by six randomly selected cultivation chambers to a total number of nine cultivation chambers. For shake flasks and bioreactors, cellular diameters were determined via CEDEX simultaneously with the cell density and viability measurements.

### Fluorescence Analysis

eGFP fluorescence was analyzed by flow cytometry measurements for bioreactor and shake flask cultivation by using an S3e™ Cell Sorter (Bio-Rad, Germany) applying a 488-nm laser in combination with a 525/30-nm filter. The obtained data were analyzed and visualized using FlowJo (Becton, Dickinson and Company, United States). For microfluidic cultivation, fluorescence microscopy was utilized with an exposure time of 20 ms and 10% intensity. Subsequently, 16-bit TIFF images for relevant time points were created to ensure maximal information density by the amount of gray scale values, and a cell’s mean gray scale value was determined manually as arbitrary unit using ImageJ 1.52p (Schindelin et al., 2012) to describe the cellular fluorescence level.

## Results and Discussion

### Key Characteristics of Stirred Tank Bioreactor, Shake Flask, and MSCC

**FIGURE 1:**
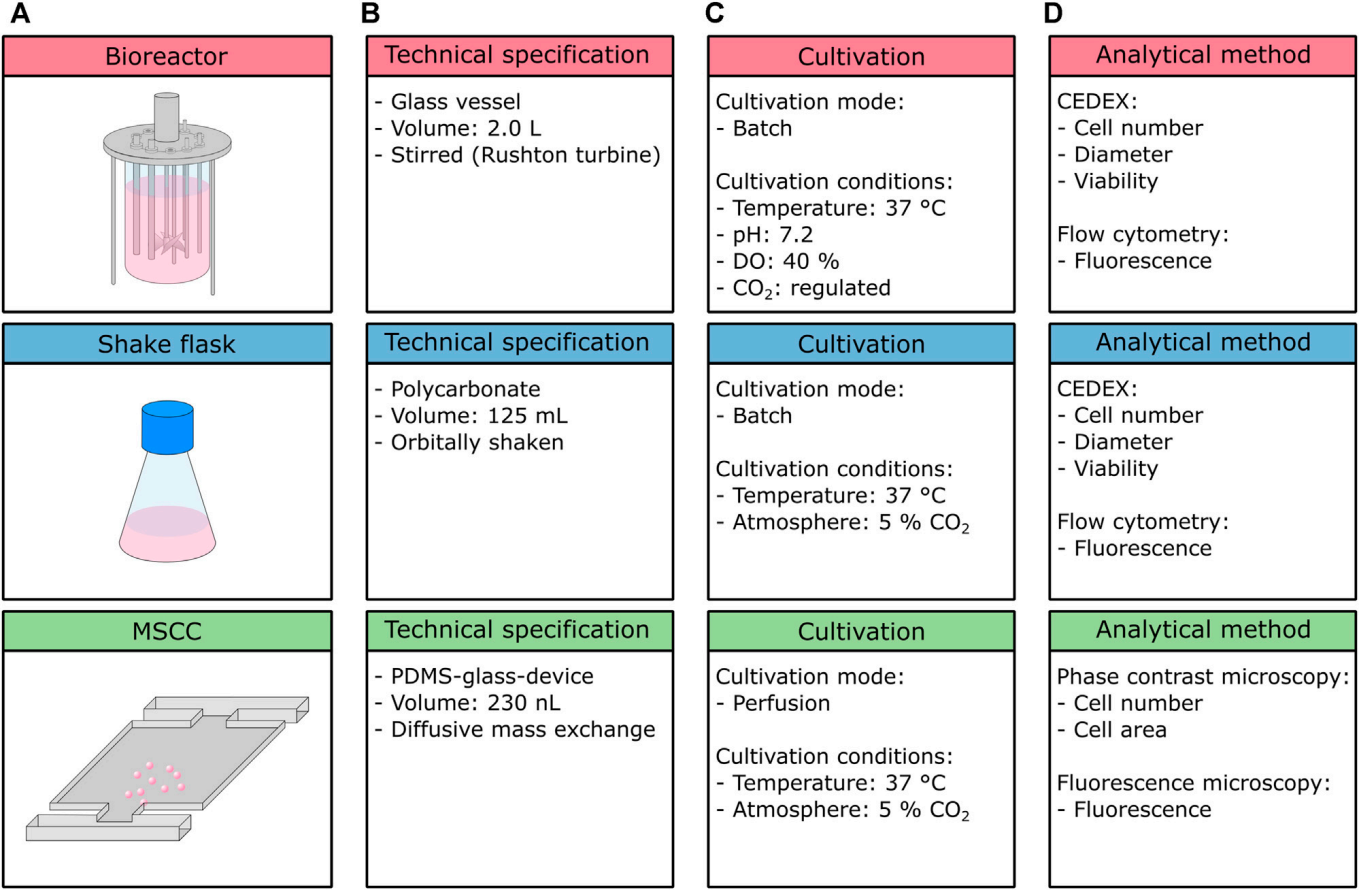
Overview of the cultivation setups used within this study. **(A)** Schematic figure of the cultivation setups, **(B)** technical specifications, **(C)** cultivation conditions and mode, and **(D)** analytical methods.

In the presented study, the growth characteristics of CHO-K1 cells in three different cultivation setups, namely, the stirred tank bioreactor, shake flask, and microfluidic single-cell cultivation device (Figure 1A) were compared. Besides the variance in cultivation volume ranging from nanoliter to liter, the cultivation setups used within this study differ in their material and their mixing approach (Figure 1B). While the bioreactor utilized here consists of a glass vessel, the shake flask is made of plastics (polycarbonate), and the applied microfluidic device is a hybrid of glass and PDMS. Therefore, cells experience different surface interactions, which might influence their physiology and thus growth behavior. Likewise, mixing is achieved by different mechanisms, namely, stirring (bioreactor), shaking (flask), and diffusion (MSCC), influencing respective mixing time, availability of oxygen, and hydrodynamic forces. Since the microfluidic device is operated under steady unaltered medium perfusion, environmental conditions can be assumed to be constant and gradients can be neglected. Because of the material used and the device’s tangential design, no limitations in oxygen concentration arise and any shear stress is minimized (Schmitz et al., 2021). In comparison, for a stirred tank bioreactor, mixing times around 10 and 100 s can be assumed but clearly depend on volume, impeller geometry, and speed (Barrett et al., 2010; Platas Barradas et al., 2012). In larger scales, temporal gradients in pH, dissolved oxygen, and dissolved CO_2_ are expectable, yet increased shear stress due to stirring and aeration might already occur in laboratory-scale stirred tank reactors. For shake flask cultivation, comparable mixing times of 5–20 s can be expected (Tan et al., 2011; Platas et al., 2013). In terms of oxygen availability, especially shaking frequency, maximal deflection, filling volume, and flask size clearly affect the oxygen transfer rate (Jänicke et al., 2007).

Bioreactor and shake flask cultivation were executed in a batch mode, whereas MSCC is performed as perfusion, thus ensuring constant environmental conditions over the whole cultivation course (Figure 1C). Due to integrated process analytical technology, cultivation conditions inside the bioreactor are feedback-regulated, while conditions inside the shake flask and microfluidic device are not subjected to any active control loops, besides a constant cultivation temperature and CO_2_ atmosphere.

On the analytical level (Figure 1D), the bioreactor and shake flask again share the same procedures: cell number for growth analysis, and diameter examination to address cellular morphology are performed off-line by application of a CEDEX cell counter. Additionally, the viability of the analyzed sample can be detected. To investigate the production behavior, here in the form of eGFP fluorescence, flow cytometry measurements are conducted. In contrast, for microfluidic cultivation cell number and cellular area are determined from phase contrast microscopy, and the fluorescence level from fluorescence microscopy. Viability cannot be determined precisely in MSCC since distinguishing between viable and dead cells is only possible if cell death has a noticeable influence on morphology, which only occurs unreliably for the cultivated CHO-K1 cells. In general, but not applied in this study, both the bioreactor and shake flask cultivation allow for more detailed metabolic analysis and thus determination of important key performance indicators such as product titer (g/l), specific productivity (pg/cell/day), nutrient consumption rates (g/cell/day), and the cumulative integral of viable cell concentration (cells/ml/day). By sampling and offline detection of, for example, amino acid profiles, glucose, or lactate concentrations, as well as antibody concentrations, the bioprocess can be described in more detail. Since MSCC allows only microscopic analysis, these parameters are not accessible, unless coupled to optical readouts like fluorescence sensors. Prospectively, the integration of novel analytical procedures like single-cell mass spectrometry will make kinetics of biomass and product formation as well as substrate uptake and consumption are determinable (Dusny, 2020).

### Comparison of Growth Behavior

Establishing a new miniaturization approach like MSCC requires systematic testing of growth behavior between traditional cultivation scales and miniaturized scales to prove its comparability. Therefore, we analyzed the growth of CHO-K1 cells with a focus on growth progression and µ_max_. The following experiments were all realized with identical cultivation medium, starting from the same master cell bank, and were inoculated after a uniform pre-culture proceeding to guarantee comparability between different scales.

**FIGURE 2:**
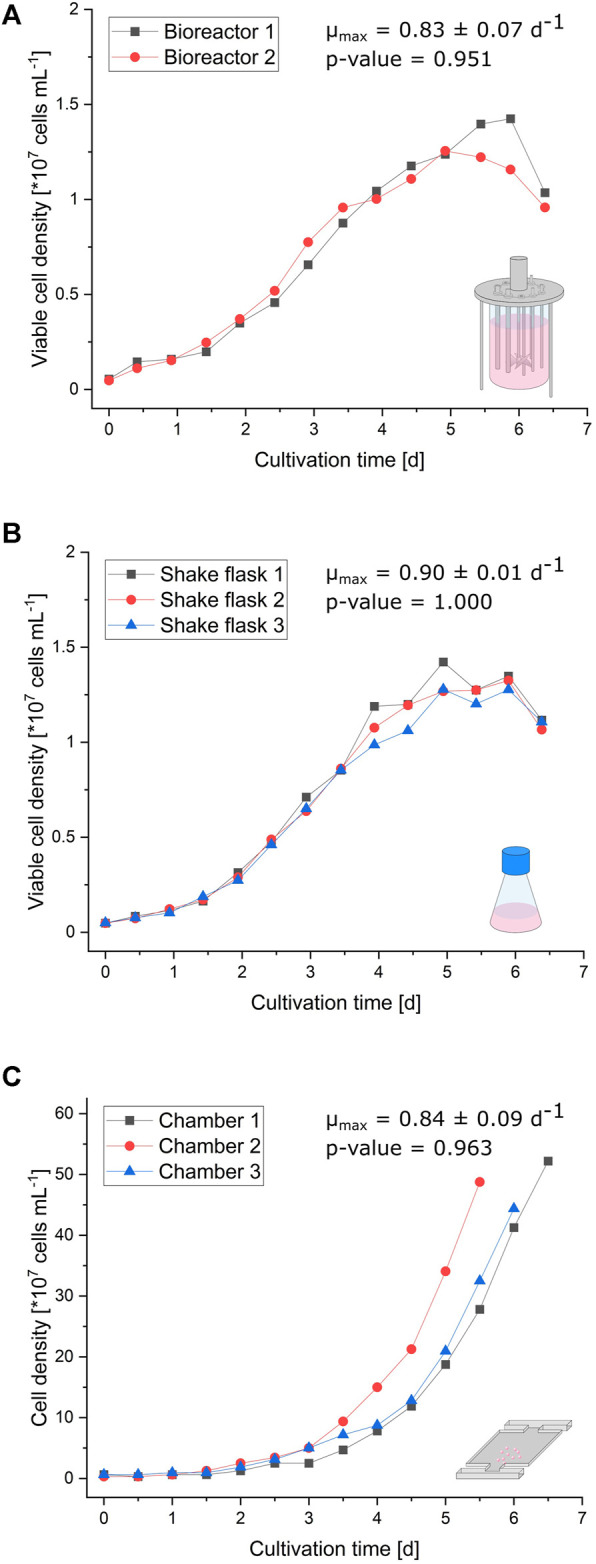
Growth comparison of the different cultivation scales. **(A)** Viable cell density of two parallel bioreactors plotted against the cultivation time. **(B)** Viable cell density of three parallel shake flasks plotted against the cultivation time. **(C)** Cell density of the three cultivation chambers plotted against the cultivation time.

The curve progressions of the (viable) cell densities illustrated in Figure 2 are very similar between the bioreactor, shake flask, and microfluidic device. Initial exponential growth from inoculation until 3 days of cultivation resembles each other in appearance. Since shake flask and bioreactor cultivations were performed in a batch mode, the growth rate declines after exponential growth phase (Supplementary Figure S2). In contrast, the growth behavior of cells cultivated in the microfluidic device stays constantly exponential until the cultivation chamber is entirely filled, due to optimal nutrient supply owing to continuous medium perfusion (Supplementary Figure S2; Supplementary Video S1). Considering the limited cultivation chamber volume, these micropopulations reach about 30-times higher maximal cell densities with approximately 4.5 × 10^8^ cells/ml than bioreactor and shake flask cultivation. The supplemental analysis of cell viability during bioreactor and shake flask cultivation reveals no significant deviation from expected trends (Supplementary Figure S3). Comparing respective maximal specific growth rates ranging from 0.83 ± 0.07 d^−1^ in bioreactors, over 0.84 ± 0.09 d^−1^ with MSCC, to 0.90 ± 0.01 d^−1^ in shake flasks also indicates a reliable comparability of miniaturized CHO-K1 cultivation and classic cultivation in milliliter and liter scales.

### Comparison of Cellular Morphology

Besides the growth behavior, cellular morphology during MSCC also needs to be validated regarding the shake flask and bioreactor. As a reference value, single-cell diameter and its distribution within the analyzed sample were investigated. Considering possible morphological changes over the course of cultivation, cell diameter distribution was measured at three time points during bioreactor and shake flask cultivation (Figure 2): straight after inoculation (*t* = 0 days), after three days (*t* = 3 days) at the end of exponential growth phase, and after 5 days (*t* = 5 days) at the beginning of stationary phase.

**FIGURE 3:**
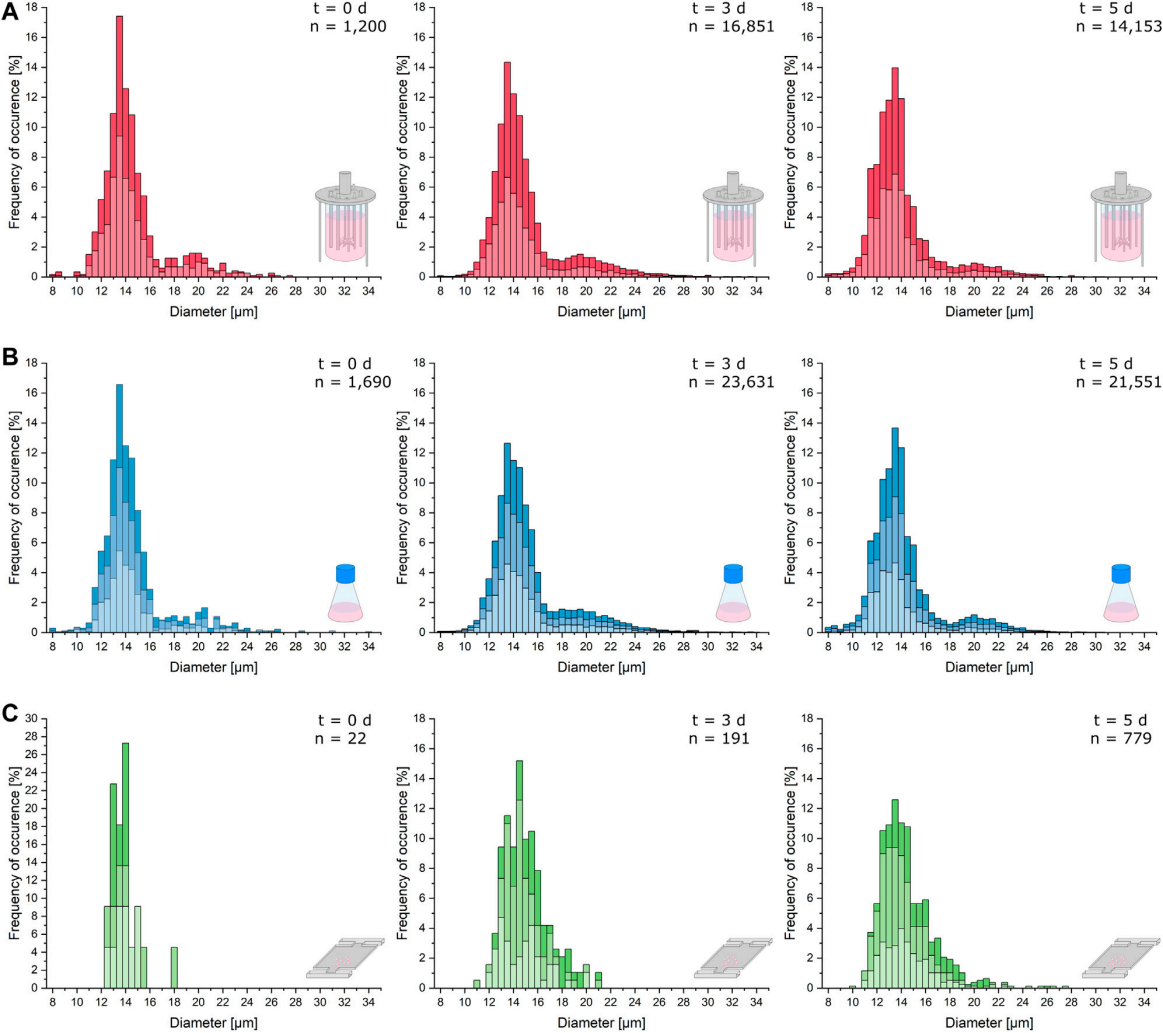
Cell morphology comparison of the different cultivation scales. Relevant cellular diameters are plotted against the frequency of their occurrence cumulated from the respective replicates of the analyzed samples. **(A)** Cell diameter distribution of the bioreactor cultivation right after inoculation (*t* = 0 days), after 3 days of cultivation (*t* = 3 days), and after 5 days (*t* = 5 days). **(B)** Cell diameter distribution of the shake flask cultivation right after inoculation (*t* = 0 days), after 3 days of cultivation (*t* = 3 days), and after 5 days (*t* = 5 days). **(C)** Cell diameter distribution of the microfluidic cultivation right after inoculation (*t* = 0 days), after 3 days of cultivation (*t* = 3 days), and after 5 days (*t* = 5 days).

As can be seen in Figure 3, the cellular diameters of the analyzed samples from MSCC, shake flask, and bioreactor show nearly identical distributions with a main peak around 14 µm. However, due to the relatively small sample size for microfluidic data at *t* = 0 days, resulting from seeding only one to three cells to every cultivation chamber, the statistical significance of the illustrated distributions from the shake flask and bioreactor is more reliable (Figure 3, left).

After 3 days of cultivation, the distributions of single-cell diameters from the bioreactor and shake flask show more cells with a diameter above 18 µm than the microfluidic cultivation (Figure 3, middle). For the shake flask and bioreactor, the distribution of bigger cells becomes more uniform than the inoculum.

After 5 days, the distribution of the microfluidic cultivation narrows further around a mean cell diameter of 14 µm with a plateau at diameters between 14.5 and 16 µm. In the shake flask, more cells with a diameter below 11 µm can be observed, and the overall distribution slightly hints at a second less dominant peak with cell diameters between 19 and 22 µm (Figure 3B, right). The same process can be hypothesized for the microfluidic cultivation (Figure 3C, right). The diameter data of the bioreactor show the same characteristics as the data obtained from shake flask cultivation (Figure 3A, right). In comparison to the distribution from the exponential growth phase, the portion of cells with a diameter over 18 µm decreases.

In general, cellular diameter data obtained from MSCC show the same distribution and trends over the course of cultivation as diameters determined from shake flask and bioreactor cultivations with only minor differences. However, the number of analyzed cells per sample for MSCC is much lower than that for shake flask and bioreactor analyses. Therefore, drawing exact conclusions concerning, for example, the population mean diameter from the displayed distribution might be prone to overinterpretation. Yet, based on the last sample time and its comparatively higher data availability, the limited cultivation chamber height of the microfluidic device seems not to influence the morphological characteristics of the analyzed cells. The analysis of the cell’s growth behavior already showed that the growth rate is not affected by the restricted device dimensions. Morphologically, a potential shift and thereby adaptive behavior over the cultivation’s course from normal diameters around 12–14 µm to a diameter of 8 µm equaling the cultivation chamber height is not noticeable.

### Green Fluorescent Protein Production

The most important parameter within bioproduction represents the cell’s productivity. As model product, eGFP was chosen to analyze cellular productivity. Therefore, an eGFP-synthesizing CHO-K1 cell pool was cultivated following the same protocols already established for the previous experiments.

**FIGURE 4:**
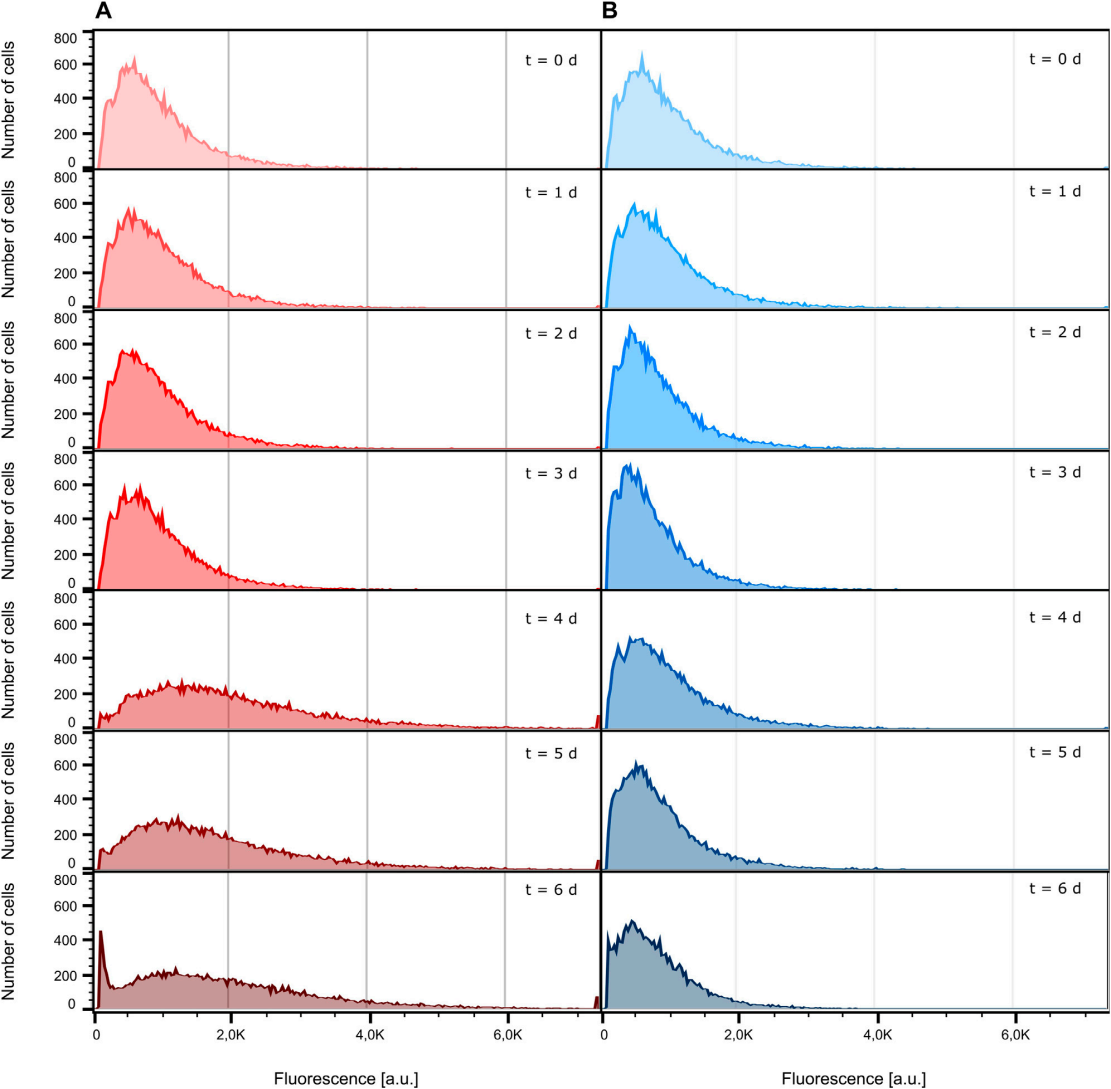
Comparison of eGFP fluorescence development. For illustrative reasons, cells identified as non-fluorescent are not displayed. **(A)** Fluorescence intensity distribution inside the bioreactor at different sampling times. **(B)** Fluorescence intensity distribution inside the shake flask at different sampling times.

Conventionally, the fluorescence behavior of cells cultivated in a bioreactor and a shake flask is analyzed by flow cytometry. For this reason, we determined the fluorescence signal of bioreactor and shake flask samples, taken every 24 h, with the help of an S3e™ Cell Sorter. Figure 4 shows the distribution of fluorescence intensity of the analyzed cells ranging from inoculation to the death phase after 6 days of cultivation (see Supplementary Figure S4 for growth curves). As can be seen, both the bioreactor and shake flask show a broad distribution of fluorescent cells. For illustrative reasons, non-fluorescent cells, which are present at any time during cultivation, are not displayed here but can be seen in Supplementary Figure S5. Corresponding values are listed in Supplementary Table S1. For the data obtained from the bioreactor, a clear tendency toward higher fluorescence intensities can be seen (Figure 4A; Supplementary Table S1; Supplementary Figure S7). In general, the whole distribution gets broader and shifts toward higher fluorescence intensities. During shake flask cultivation, the fluorescence distribution of the analyzed cells does not change its character as observed for the bioreactor cultivation (Figure 4B) and the mean fluorescence intensity stays unchanged (Supplementary Table S1; Supplementary Figure S6). For both cultivation methods, the ratio of fluorescent to non-fluorescent cells remains constant for the first 4 days of cultivation. After 5 days, the portion of non-fluorescent cells increases throughout the analyzed samples (Supplementary Table S1; Supplementary Figure S6).

The data presented in Figure 4 only show the population’s status at one specific time point during the cultivation. Therefore, these measurements are limited to population dynamics between distinct sampling times and do not yield any insights into dynamic fluorescence development of single cells. Furthermore, it is not possible to retain the same group of individual cells across the course of the cultivation using flow cytometric analysis, meaning that at every sampling time, different cells are analyzed. Yet, knowing if single cells show steadily increasing fluorescence levels, representing a constant product formation, or fluctuate in their productivity is a valuable information to classify the performance of a bioprocess. For this purpose, MSCC needs to be applied to examine single-cell dynamics.

**FIGURE 5:**
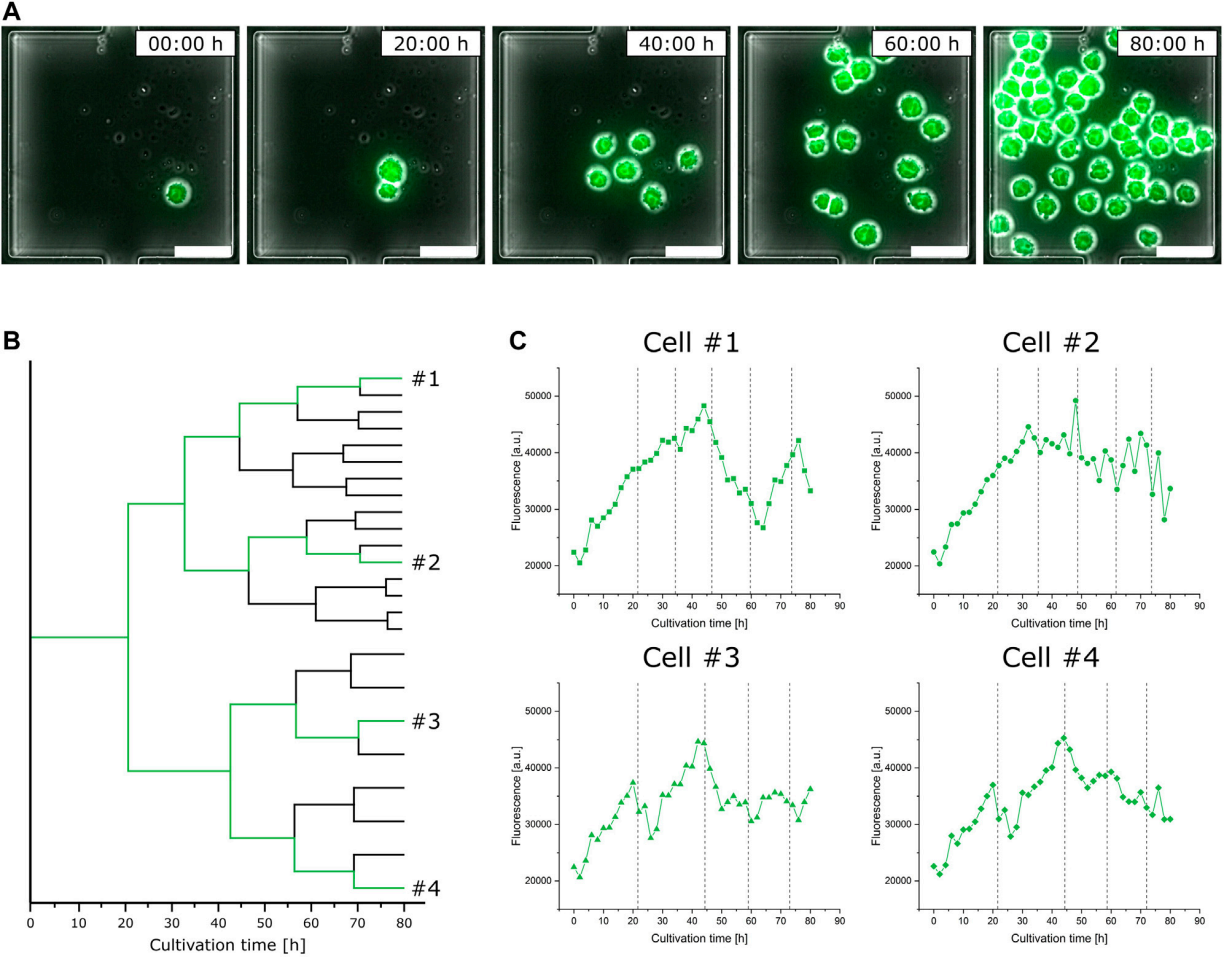
Analysis of eGFP fluorescence single-cell dynamics during microfluidic cultivation. **(A)** Time-lapse image sequence showing the growth and fluorescence development of an isogenic microcolony **(scale bar = 50 µm)**. **(B)** Lineage tree of the same isogenic microcolony. **(C)** Fluorescence development of four exemplary single cells over the cultivation time; dotted lines indicate cell division events.

In addition to the performed population analysis, which shows highly comparable characteristics in general fluorescence distribution to the data obtained from the bioreactor and shake flask via flow cytometry (Supplementary Figure S7), we investigated single-cell fluorescence development for a representative isogenic population to exemplify feasible single-cell analysis (Figure 5A). Considering the doubling time of single cells, already after the first cell division, the two originated daughter cells greatly differ in the duration until their second cell division by the factor of two (Figure 5B). Looking at the respective video (Supplementary Video S2), it appears to be very likely that this divergence arises from the asymmetrical division of the mother cell. In the following subsequent cell divisions, both daughter cell’s offspring show only little variations (Figure 5B).

This heterogeneous behavior can not only be found in relation to growth but also has a noticeable impact on single-cell fluorescence. Figure 5C shows the fluorescence development of four exemplary cells over time, each from different branches of the population’s lineage tree, with their respective cell division events marked by dotted lines. Looking at cell #1, varying stages of fluorescence increase and decrease can be identified. Although having the closest relation, the fluorescence course of cell #2 shows a slight but steady decrease and does not feature comparable fluctuating tendencies like cell #1. Comparing cell #3 against cell #4, both vary only slightly in their course after originating from a common progenitor cell.

Relating the sections of fluorescence increase and decrease from Figure 5C to the respective moments of cell division, no obvious interrelationship can be identified. Nevertheless, the already discussed asymmetrical division not only influences following cell division events but also results in a drastic fluorescence decrease.

Disregarding the differences in their individual fluorescence development, all cells in Figure 5C do not show comparable trends in fluorescence intensity to the population analysis of shake flask and bioreactor cultivation (Supplementary Figure S6). Unlike MSCC, the fluorescence development of bioreactor samples shows a clear trend toward higher mean intensities with a slight increase in the non-fluorescent portion of the analyzed population, while the fluorescence intensity of the shake flask stays at a constant level with nearly two-fold increase of non-fluorescent cells. This observation stresses the importance of dynamic single-cell analysis since detecting fluctuating production behavior represents the first step toward eventually engineering bioprocesses for higher productivities by more stable product formation behavior.

## Conclusion and Outlook

Environmental control, live cell imaging, and high spatio-temporal resolution make MSCC a highly valuable miniaturization tool as single-cell dynamics under constant cultivation conditions can be analyzed over multiple generations, and therefore, intercellular differences in growth behavior or fluorescence-coupled protein expression can be investigated. These analyses are not performable applying standard average measurements as it is common with other small-scale systems like microtiter plates or miniaturized bioreactors.

The study presented here shows that MSCC-generated data are comparable to data from laboratory-scale cultivation approaches in all investigated aspects, namely, growth, cellular morphology, and production behavior. Regarding growth behavior, cells cultivated on-chip showed a specific growth rate in the same order as populations cultivated in shake flasks or stirred tank bioreactors. Likewise, although restricted due to a limited cultivation chamber height, cellular morphology concerning the single-cell diameter of MSCC showed the same trends and mean diameters as cells that were cultivated in bigger scales. Despite the different quantification approaches, for eGFP production, the fluorescence distributions throughout the analyzed populations were comparable as well. In addition to the population dynamics investigated via flow cytometry, MSCC allowed analysis of single-cell fluorescence dynamics and revealed distinct phases of fluorescence increase and decrease. In contrast, for shake flask cultivation, a constant fluorescence intensity level can be detected over the course of cultivation, while in bioreactor cultivation, an increasing fluorescence intensity was detected. Thus, applying conventional flow cytometric analysis without temporal resolution and only looking at average fluorescence intensities over time masks the remarkable single-cell fluorescence dynamics, and thereby only permits limited or even misleading insights into the analyzed production process.

The proven comparability between the microfluidic miniaturization tool and shake flask or laboratory-scale bioreactor cultivation enables MSCC to be applied for numerous applications in basic research as well as for bioprocess development. In the context of mammalian bioproduction, especially studying cellular heterogeneity concerning growth behavior and productivity inside isogenic populations is of utmost interest since it can have a severe influence on bioprocess robustness and outcome (Paul and Herwig, 2020). Particularly, the number of generations and the resulting cellular heterogeneity, with its effects from single-cell cloning up to commercially application, represents a very important question in process development (Frye et al., 2016; Rugbjerg and Sommer, 2019), which eventually can be analyzed more closely by the means of MSCC.

## Data Availability

The raw data supporting the conclusions of this article will be made available by the authors, without undue reservation.

## Supplementary Material

Supplementary Video 1 Microfluidic single-cell cultivation of CHO K1 suspension cells.

Supplementary Video 2 eGFP production of CHO K1 suspension cells.
