# Supplementary material for: Growth and eGFP Production of CHO-K1 Suspension Cells Cultivated From Single Cell to Laboratory Scale
Source: Front Bioeng Biotechnol. 2021 Oct 15;9:716343. doi: 10.3389/fbioe.2021.716343 (PMC8554123; doi:10.3389/fbioe.2021.716343)
Supplement: Supplementary file 3 [file DataSheet1.docx]

***Supplementary Material***


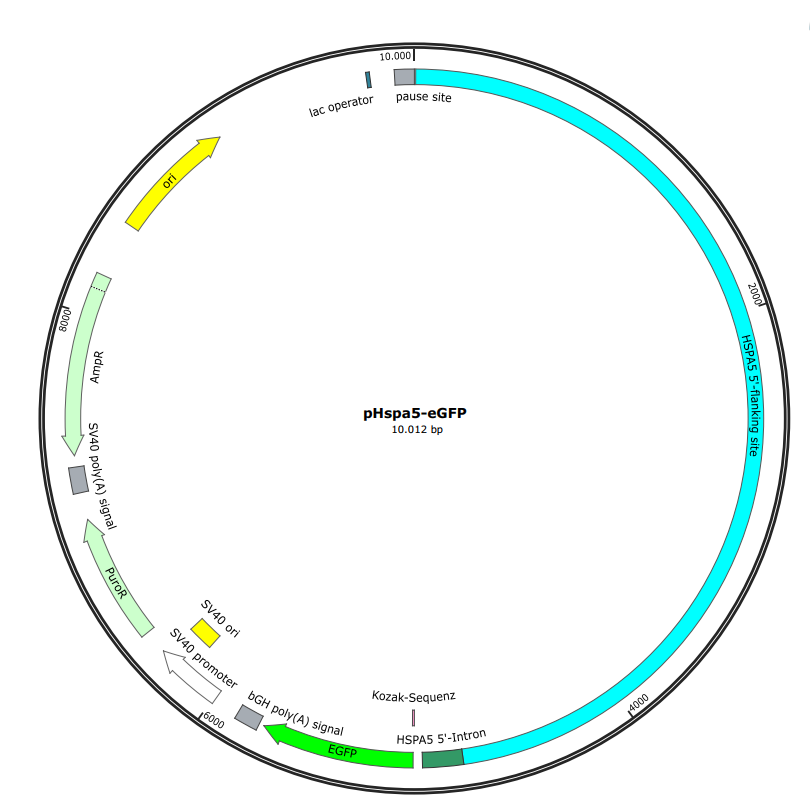


**Supplementary Figure 1.** Transfection vector showing the endogenous HSPA5 promoter and eGFP gene as well as a puromycin resistance for selection. The map was created using SnapGene® (GSL Biotech LLC, USA).


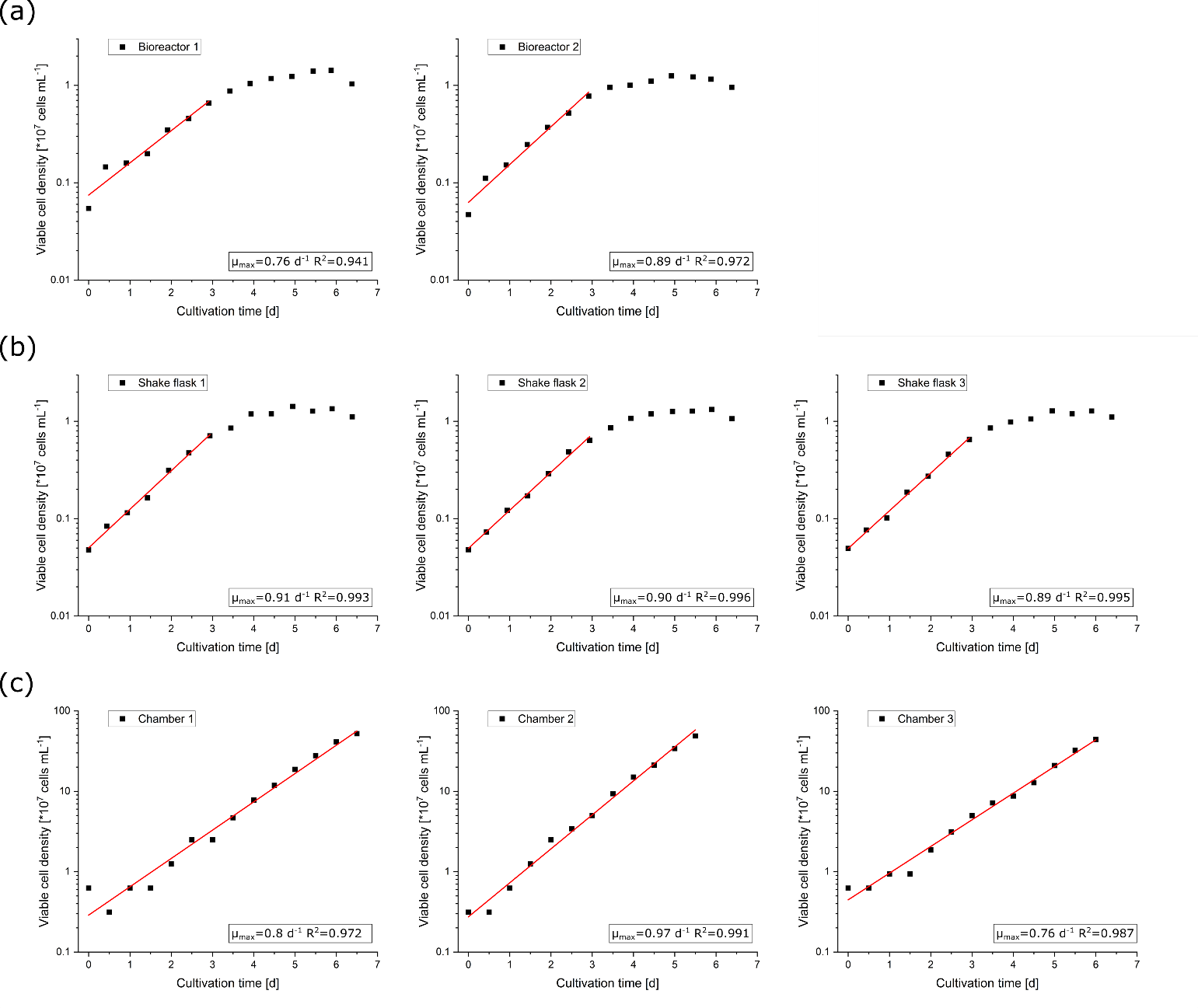


**Supplementary Figure 2.** Semi-logarithmically plotted growth profile of the (a) bioreactors, (b) shake flasks, and (c) MSCCs with their resulting linear fit in exponential growth phase. For every colony the graphically determined growth rate µ with its coefficient of determination R^2^ are placed in the respective plot at the bottom right.


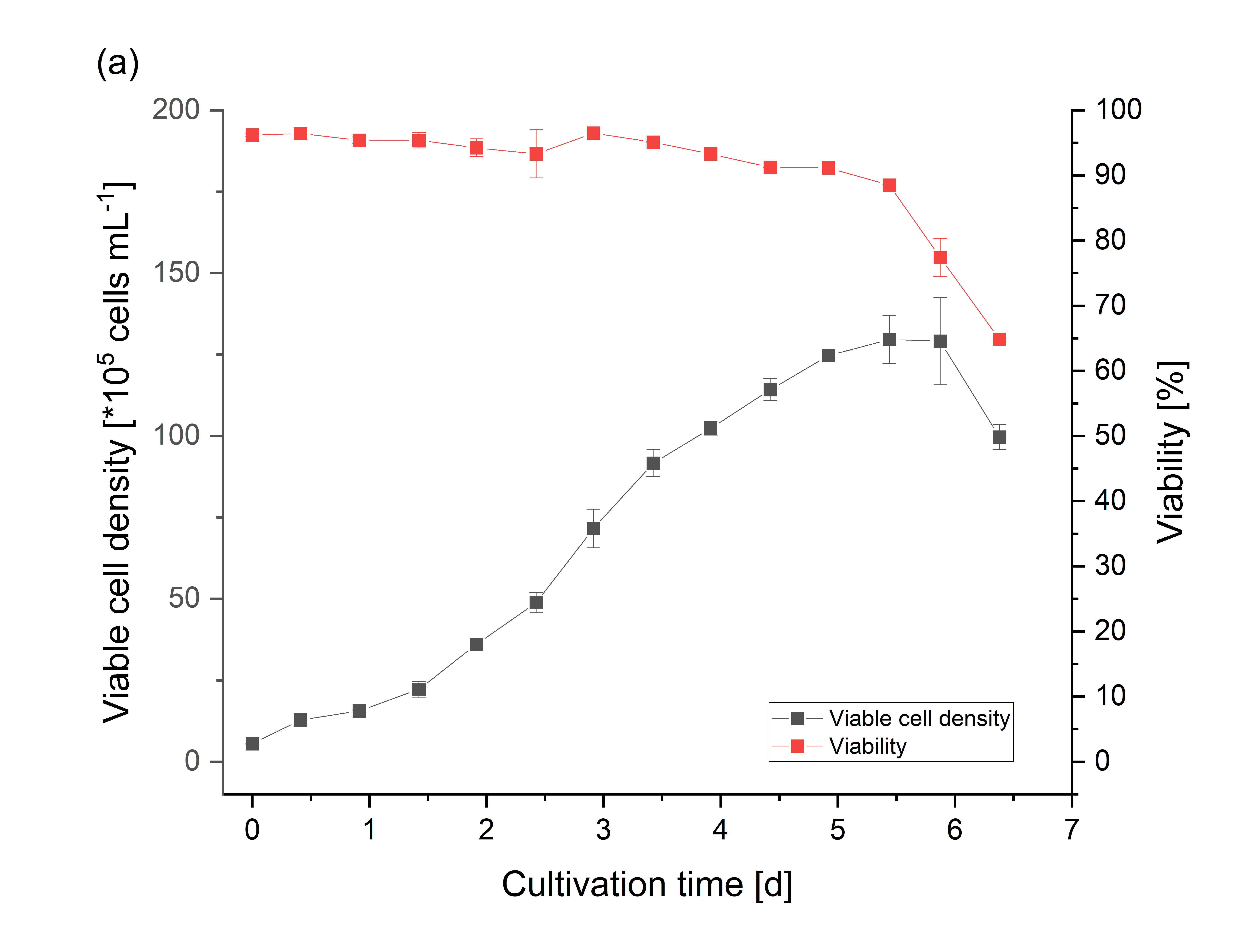

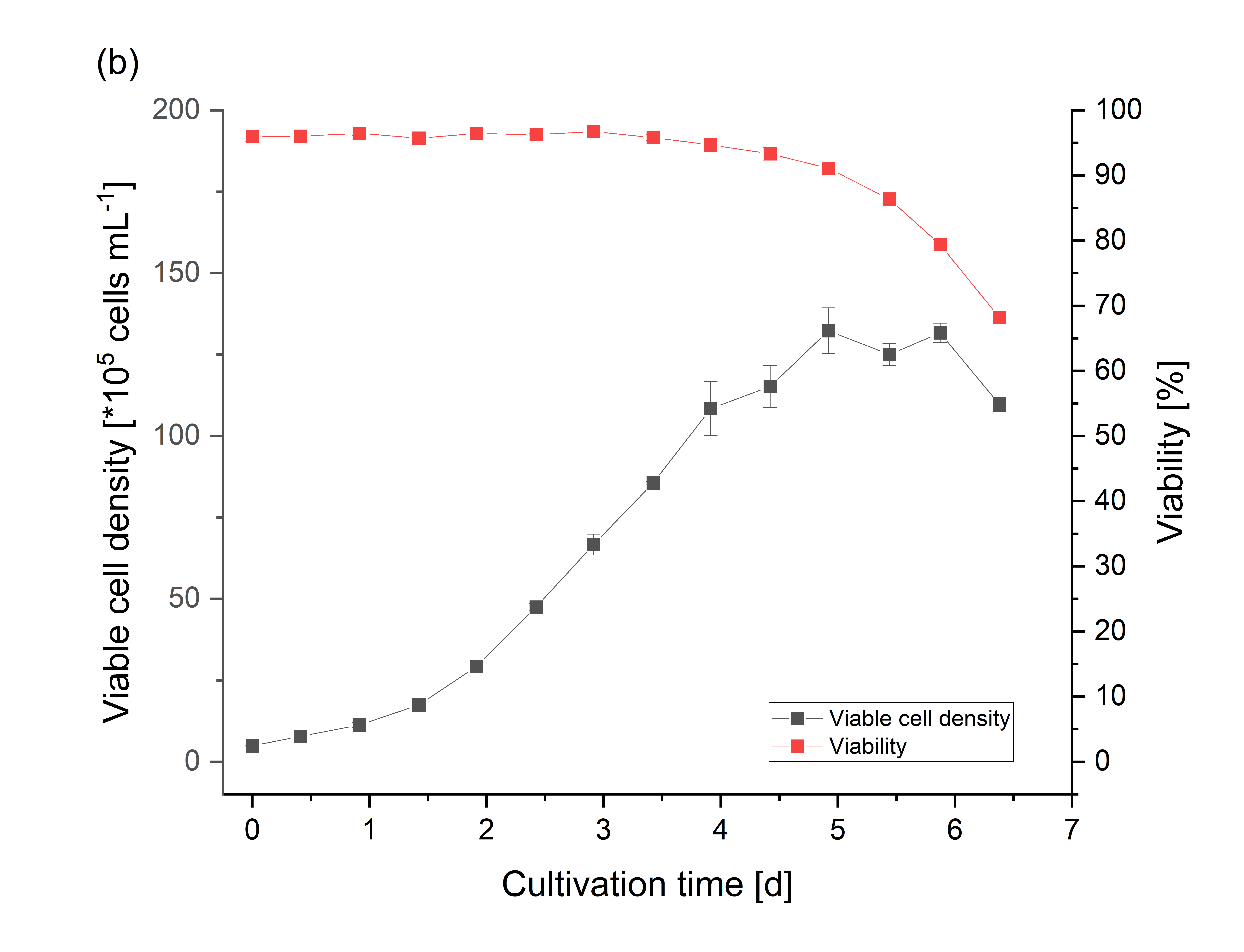


**Supplementary Figure 3.** Viable cell density and viability profile of the (a) bioreactor and (b) shake flask cultivation. Both viable cell density and viability are averaged for the respective replicates (bioreactor n_cultivation_ = 2, shake flask n_cultivation_ = 3).


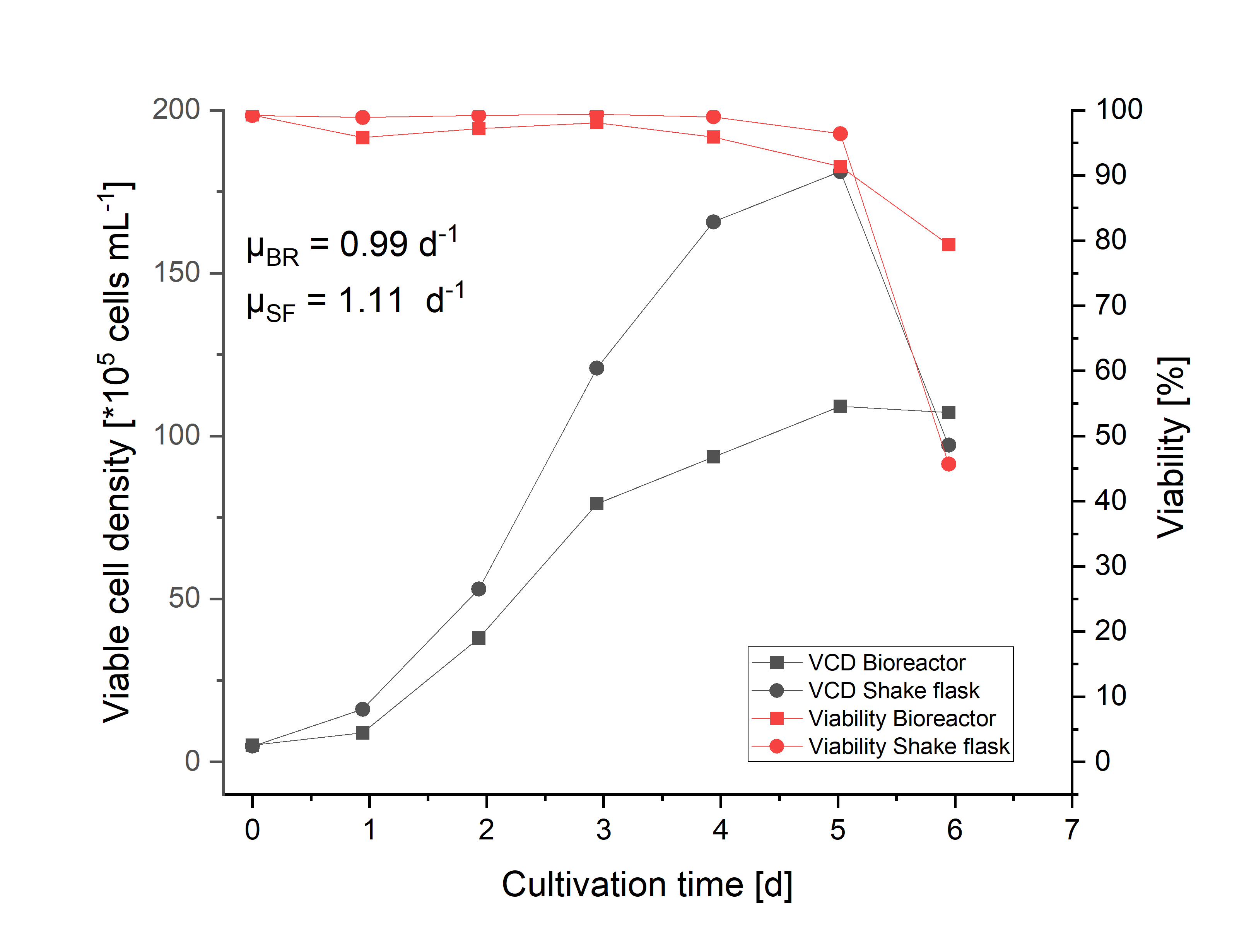


**Supplementary Figure 4.** Viable cell density (VCD) for bioreactor and shake flask cultivation of the eGFP-producing CHO-K1 cell pool.


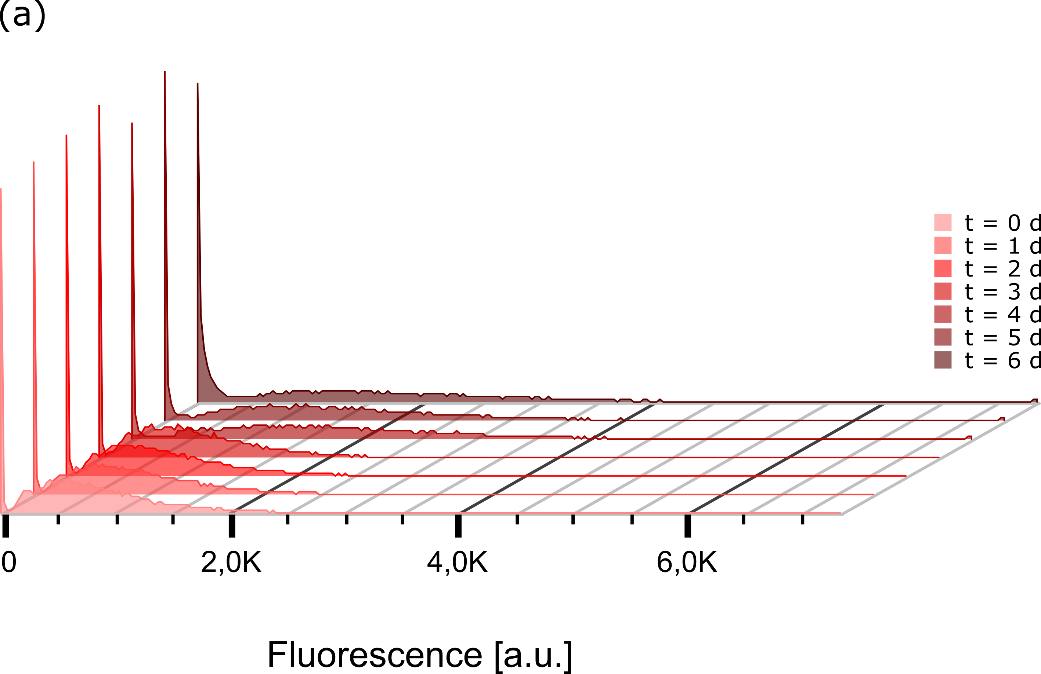

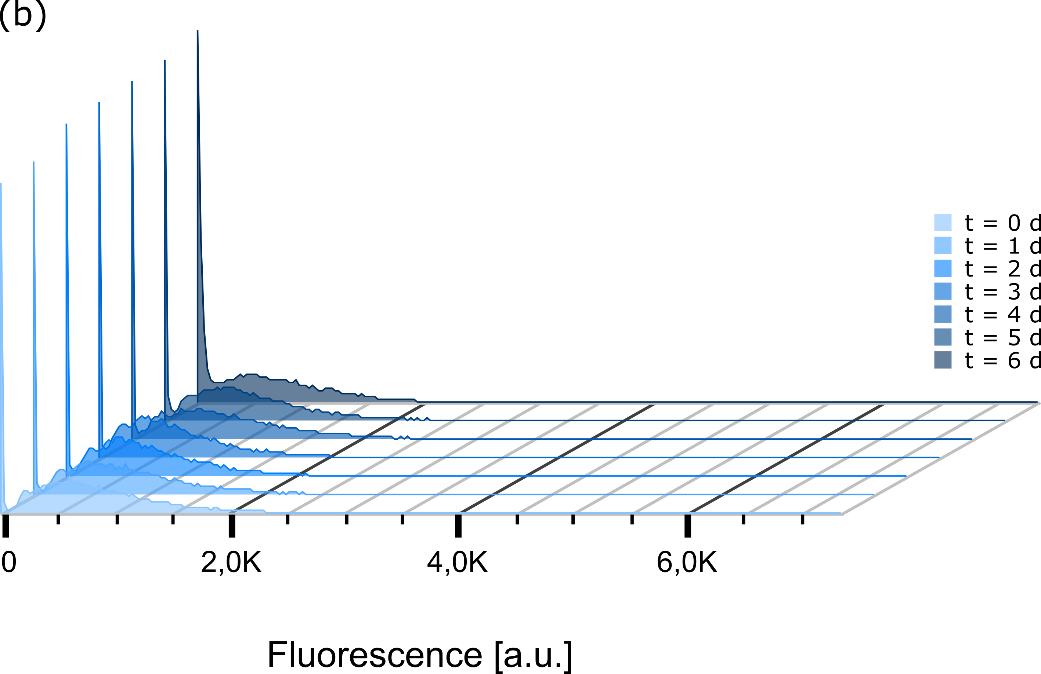


**Supplementary Figure 5.** eGFP distribution at different sampling times during (a) bioreactor and (b) shake flask cultivation. Displayed are both fluorescent and non-fluorescent cells of the respective sample.

**Supplementary Table 1.** Summary of the flow cytometric analysis of the eGFP-production from shake flask and bioreactor illustrated in Fig. S6. For every sampling time the number of analyzed cells, the percentage of non-fluorescent cells, the percentage of fluorescent cells, and the median of the fluorescence intensities are displayed.

| **Sampling time** | **Parameter** | **Shake flask** | | **Bioreactor** | |
| --- | --- | --- | --- | --- | --- |
|  |  | **Percentage / %** | **Cells / -** | **Percentage / %** | **Cells / -** |
| **t0** | **Singularized** | 99.4 | 29830 | 99.6 | 29874 |
|  | **eGFP negative** | 20.8 | 6194 | 20.4 | 6099 |
|  | **eGFP positive** | 79.2 | 23633 | 79.6 | 23770 |
|  | **Median of fluorescence [a.u.]** |  | 804 |  | 799 |
|  |  |  |  |  |  |
| **t1** | **Singularized** | 99.7 | 29899 | 99.5 | 29864 |
|  | **eGFP negative** | 21 | 6272 | 21.4 | 6386 |
|  | **eGFP positive** | 79 | 23627 | 78.6 | 23479 |
|  | **Median of fluorescence [a.u.]** |  | 799 |  | 858 |
|  |  |  |  |  |  |
| **t2** | **Singularized** | 99.8 | 29937 | 99.7 | 29915 |
|  | **eGFP negative** | 22.3 | 6674 | 22 | 6578 |
|  | **eGFP positive** | 77.7 | 23265 | 78 | 23338 |
|  | **Median of fluorescence [a.u.]** |  | 704 |  | 819 |
|  |  |  |  |  |  |
| **t3** | **Singularized** | 99.8 | 29947 | 99.9 | 29957 |
|  | **eGFP negative** | 22.9 | 6848 | 22.4 | 6725 |
|  | **eGFP positive** | 77.1 | 23100 | 77.6 | 23234 |
|  | **Median of fluorescence [a.u.]** |  | 638 |  | 817 |
|  |  |  |  |  |  |
| **t4** | **Singularized** | 99.8 | 29951 | 97.7 | 29314 |
|  | **eGFP negative** | 22.9 | 6863 | 21.5 | 6314 |
|  | **eGFP positive** | 77.1 | 23088 | 78.5 | 23000 |
|  | **Median of fluorescence [a.u.]** |  | 822 |  | 1744 |
|  |  |  |  |  |  |
| **t5** | **Singularized** | 99.9 | 29974 | 99.7 | 29910 |
|  | **eGFP negative** | 24.1 | 7213 | 24.6 | 7345 |
|  | **eGFP positive** | 75.9 | 22762 | 75.4 | 22566 |
|  | **Median of fluorescence [a.u.]** |  | 727 |  | 1528 |
|  |  |  |  |  |  |
| **t6** | **Singularized** | 99.9 | 29957 | 99.7 | 29917 |
|  | **eGFP negative** | 37.8 | 11309 | 29.3 | 8768 |
|  | **eGFP positive** | 62.3 | 18649 | 70.7 | 21153 |
|  | **Median of fluorescence [a.u.]** |  | 706 |  | 1664 |

**
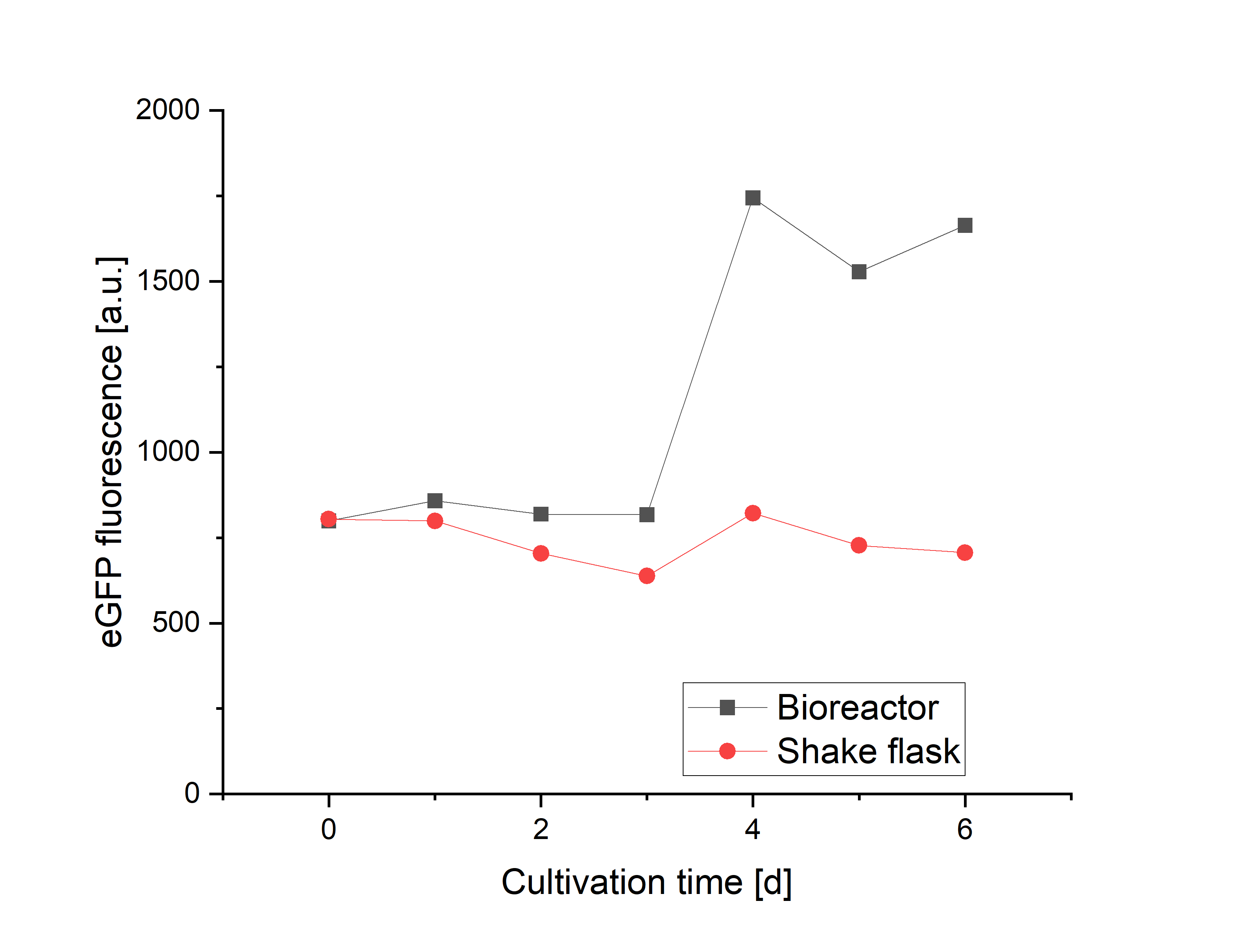

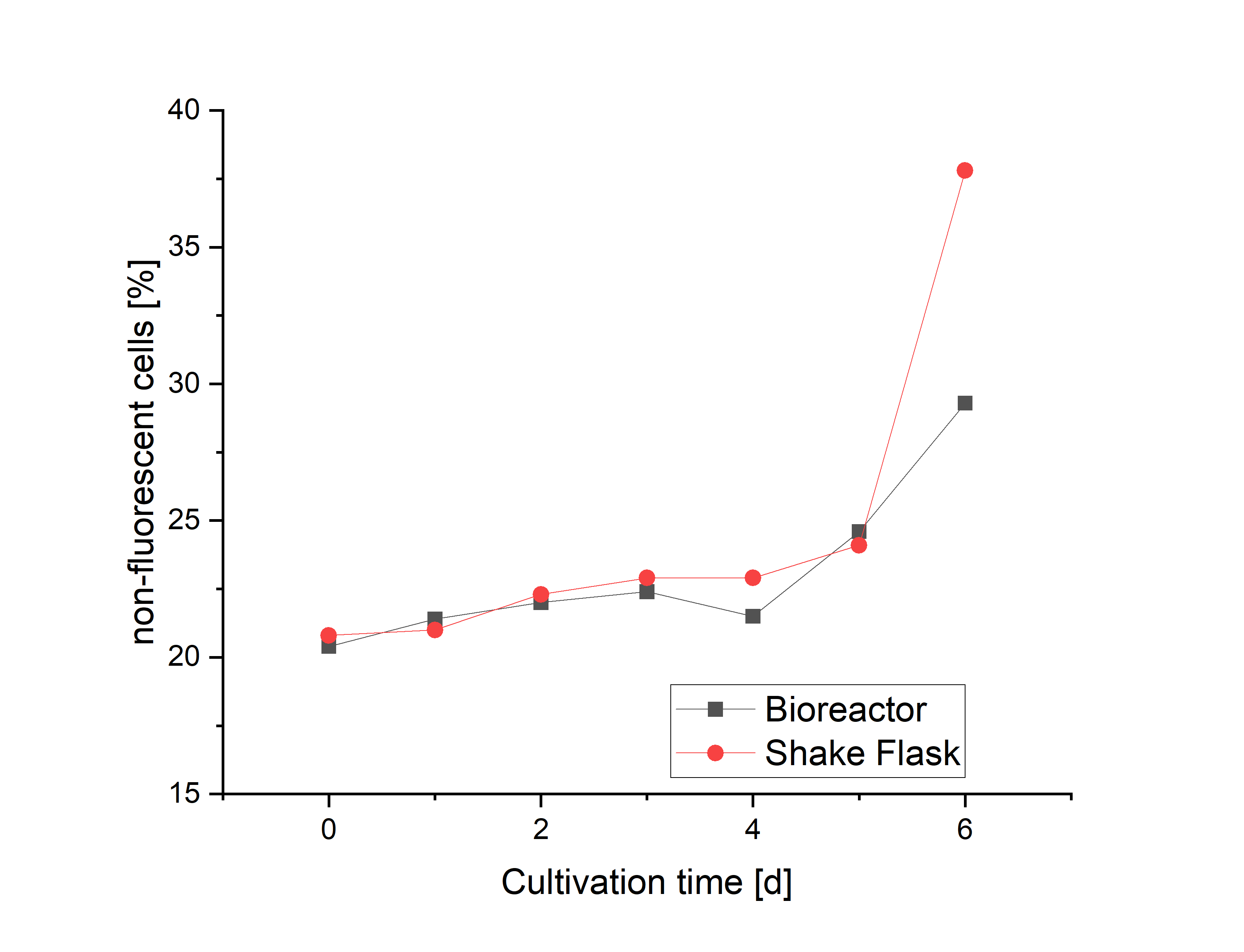
**

**Supplementary Figure 6.** Average eGFP level as well as portion of non-fluorescent cells of bioreactor and shake flask samples over the cultivation time.


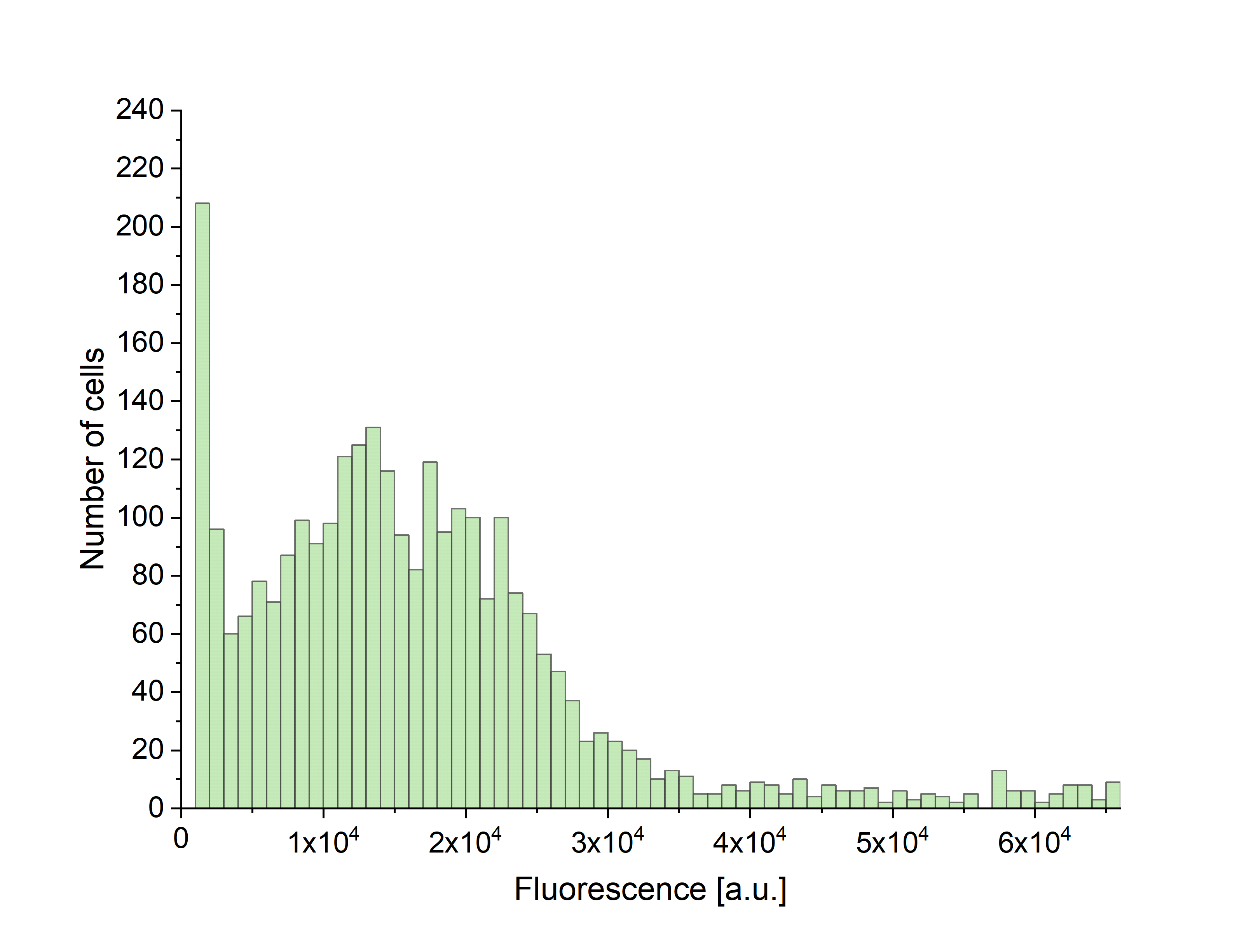


**Supplementary Figure 7.** eGFP distribution of 44 analyzed microcolonies after 80 h (during exponential growth) of microfluidic single-cell cultivation (n_cells_ = 2,800).
